# The Relationship between Personality Traits and COVID-19 Anxiety: A Mediating Model

**DOI:** 10.3390/bs12020024

**Published:** 2022-01-26

**Authors:** V. Vineeth Kumar, Geetika Tankha

**Affiliations:** Department of Psychology, Manipal University Jaipur, Jaipur 303007, India; geetika.tankha@jaipur.manipal.edu

**Keywords:** PAS-10, fear, somatic concern, COVID-19, personality traits, neuroticism

## Abstract

The COVID-19 pandemic has created a lot of fear and anxiety globally. The current study attempted to investigate the association among the big five personality traits and the two factors of COVID-19 pandemic anxiety (fear and somatic concern). Further, sleep quality as a mediator between personality traits and pandemic anxiety was also assessed. The study involved a cross-sectional sample of 296 adult Indians who were administered the 10-item short version of BFI along with the COVID-19 Pandemic Anxiety Scale and Sleep Quality Scale. Path analysis was used to test the theoretical model that we proposed. The overall model has explained 6% and 36% of the variance, respectively, for the factors of fear and somatic concern of COVID-19 pandemic anxiety. The path analysis model indicated that only the trait of neuroticism showed a significant direct and indirect effect on pandemic anxiety in the sample. Those scoring high on neuroticism indicated high levels of fear as well as somatic concern. Neuroticism also showed partial mediation through sleep quality on the factor of somatic concern. Agreeableness was the only other personality trait that indicated a significantly negative relationship with the factor of somatic concern. These relationships were independent of age, gender, and occupational status. These findings provide a preliminary insight into the slightly different relationship which has emerged between personality and COVID-19 pandemic anxiety in comparison to general anxiety.

## 1. Introduction

The COVID-19 pandemic has threatened the lives of human beings and has impacted every aspect of human life. The pandemic has affected those infected by the virus and those who were not. The life-threatening nature of the virus combined with an uncertain environment has created anxiety among the masses. It has altered the way people work, travel, communicate, and live their everyday lives. Any disruption of this kind can have an adverse psychological impact on individuals. Being anxious is part of human nature. However, when the anxiety level goes high and becomes chronic, it may adversely impact an individual’s well-being. Most of the time, for individuals suffering from general anxiety, the cause is unknown. One of the differentiating factors between general anxiety and pandemic anxiety is that the cause of worry points to the pandemic (e.g., COVID-19) for human uneasiness and worry.

It seems COVID-19 pandemic anxiety stems from two components, i.e., fear and somatic concern [1]. First, continuous information overload related to the virus spreads triggered fear in individuals. People feared that if the virus infected them, it could lead to a painful death. It led them to be wary of going out to public places or meeting people. They started isolating themselves. Lockdowns and restrictions to move around added more fear to the tense environment. The need to continuously check and monitor any symptoms related to the pandemic created pressure and disrupted the daily functioning of individuals. The insecurity due to the lack of control over the situation and subsequent overindulgence in performing safety behaviors have also enhanced fear. Second, any slight perceived physiological symptoms like palpitations, shortness of breath and giddiness, or changes in eating and sleep patterns made people hyper-vigilant and concerned that they may have the coronavirus. Thus, the anxiety-driven apprehensions made people dysfunctional at psychological and social levels. With the spread of coronavirus across the world, this phenomenon of COVID-19 anxiety became more evident. Studies have demonstrated that the COVID-19 pandemic has had a severe negative bearing on mental health [2] and the psychological well-being of people across the world [3].

Since the onset of the current COVID-19 pandemic, it has been observed that the number of COVID-19 cases across nations keeps fluctuating. The virus is mutating, and the medical fraternity finds it quite challenging to make any concrete prediction regarding the end of the current pandemic. As the pandemic has been ravaging the planet for the last two years and with no end in sight, people can develop a sort of pandemic fatigue. This fatigue can prevent people from taking and maintaining precautionary measures against COVID-19. The probability of the current pandemic becoming an endemic also seems a possibility. The mental health challenges among the masses during the current pandemic can be pretty severe. Thus, identifying behavior that can help alleviate the mental health burden on individuals is quite significant. A highly anxious individual often finds it challenging to analyze, face, and successfully navigate challenging problems or situations. Somehow, the current COVID-19 pandemic has become a challenge for everyone. Identifying psychological variables that can help mitigate the mental health burdens of individuals can be beneficial in this scenario. An individual’s personality plays an essential role in facilitating or obstructing how they perceive and respond to day-to-day events in unique or critical times. Personality traits can be markers for understanding the individual differences found in the way people show concern, readiness, and adaptability to environmental tragedies and exigencies. Similarly, improving sleep quality may help reduce anxiety due to the pandemic. Understanding the relationship between the variables can enable researchers and mental health professionals to develop intervention programs to help individuals manage COVID-19 pandemic anxiety.

### 1.1. Literature Review

#### 1.1.1. Big Five Personality Traits and COVID-19 Anxiety

To a great extent, personality traits guide and shape an individual’s responses to life events through their thoughts, emotions, and behavioral actions [4,5]. For example, individuals high on agreeableness and extraversion traits experience positive emotions. They also positively evaluate their daily activities [6]. In a study on students, openness to experience has been associated with coronavirus anxiety. However, there was a negative relation between conscientiousness with pandemic-related stress [7]. Thus, openness emerged as a risk factor, and conscientiousness was a protective personality factor. Another study reported a significant negative association for four of the big five traits, except neuroticism, which is positively associated with COVID-19 anxiety [8].

Research studies have substantiated that those individuals who are high on the personality trait of neuroticism could have a significantly increased risk perception of a pandemic [9,10]. Additionally, studies have indicated that neurotics have shown weaker mental well-being even in regular times, whereas extraverts have demonstrated stronger mental health [11,12,13]. It is a general belief that individuals who score highly on the trait of neuroticism report more negative rather than positive emotions. As a result, they are more susceptible to adverse outcomes when faced with traumatic experiences. These individuals are more anxious and insecure [14], predisposed to psychological distress [15], and more impulsive in their behavior in comparison to individuals who score low on neuroticism [16]. Studies have also indicated that individuals with high neuroticism scores experience higher generalized anxiety, depression [17], and negative affect [18,19]. They also experience more significant discomfort due to the work/study restrictions and a lower level of subjective well-being [20].

A recent study reported that neuroticism was positively and significantly related to high anxiety during the Covid-19 outbreak [21]. Further studies indicated that neuroticism and extroversion personality traits are strongly associated with mental health [11,12,13,15,22]. In general, the expectation is that an extravert will have better adjustment levels and a lower degree of anxiety. However, extraverts who desire higher levels of social and physical activity and interpersonal interaction could find the enforced isolation and restrictions of free movement during the current pandemic particularly difficult compared to introverts. Researchers have reported that the strict measures associated with the pandemic, like social isolation and social distancing, would be more natural for introverts than outgoing and social extraverts [23,24].

On the other hand, the expectation is that people with a high level of neuroticism might generally be less disturbed with being forced to stay at home rather than going out to face the everyday hassles of life. Nevertheless, increased uncertainty about being infected by the virus could offset this effect. Thus, it indicates that personality traits predispose an individual’s intensity and experience of anxiety.

#### 1.1.2. Big Five Personality Traits and Sleep Quality

Sleep is a common element of life and an essential quality health and well-being component. Conversely, poor sleep and disruptive sleeping patterns can lead to physical and psychological problems [25,26,27,28]. Past research has indicated that the personality trait of neuroticism is inversely related to good sleep quality [29,30,31,32,33,34,35]. On the other hand, the relation between conscientiousness and sleep is mixed. Some studies show a positive association with better quality sleep [31,32,33,35,36], while others indicate no association [29,30].

Similarly, high extraversion and low neuroticism are closely associated with better sleep [37]. Thus, the best predictor of sleep quality has been neuroticism. However, when all the predictors were put together in a single model, only agreeableness became a significant predictor of sleep quality [30]. Furthermore, sleep duration is also one of the critical indicators of good sleep quality. For example, individuals scoring high on traits of neuroticism and openness were short sleepers [38]. Thus, the past research indicates that neuroticism is outrightly inversely related to sleep quality, whereas the other four traits have a mixed or no association with sleep quality. However, these studies were done before the COVID-19 pandemic, and the studies done post-COVID-19 have focused on the clinical population [39] or frontline workers [40].

#### 1.1.3. Sleep Quality and COVID-19 Anxiety

The research studies suggest that good sleep quality and anxiety are inversely related [41,42,43,44]. A study found that students who had poor sleep the previous night reported higher anxiety levels [45]. Research has also indicated that poor sleep quality can change anxiety symptoms [46,47]. In another study on university students, perceived anxiety was negatively associated with sleep quality [48]. They also confirmed that sleep education and sleep improvement-based intervention programs could help improve students’ mental health and reduce stress and anxiety levels. A meta-analysis involving randomized controlled trials revealed that improving sleep quality had, on average, a medium-sized effect on mental health.

Furthermore, research indicates that sleep quality reduces depression, anxiety, and stress [49]. Nevertheless, a few studies have also indicated a bi-directional relationship between sleep quality and anxiety [50,51]. However, these relationships have not been explored in the context of COVID-19 anxiety. In addition, there is a paucity of research investigating the possible mediating role of sleep quality in the relationship between personality traits and COVID-19 anxiety in adults. Similarly, most contemporary researchers have focused on assessing the COVID-19 pandemic anxiety using instruments of general anxiety, e.g., Depression Anxiety and Stress-21 [52], Self-rating Anxiety Scale [53], and Generalized Anxiety Disorder 7-item [54]. However, as pandemics are rare and each pandemic is unique in nature, symptoms, duration, and impact, each pandemic has to be dealt with differently. Therefore, understanding whether the big five personality traits predict COVID-19 anxiety and exploring the role of sleep quality in mediating the relationship could assist mental health professionals in developing individually tailored guidance and interventions for managing COVID-19 anxiety.

### 1.2. The Present Study

**Figure 1:**
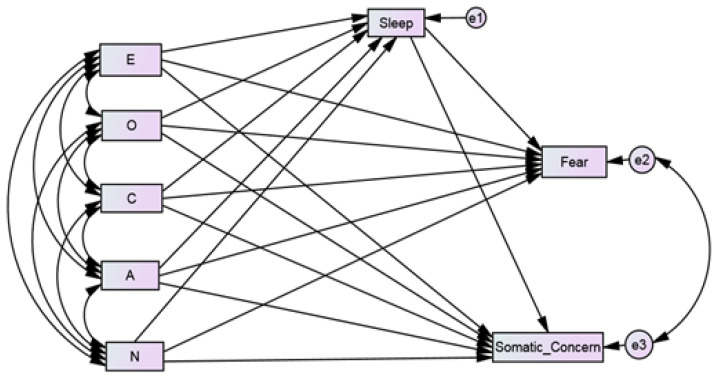
Conceptual model showing the relationship between the big five personality traits (E = Extraversion, O: Openness, C: Conscientiousness, A: Agreeableness, N: Neuroticism) and COVID-19 anxiety components (Fear and Somatic Concern) mediated through sleep quality.

The present study explores the relationship of big five personality traits with the two factors of COVID-19 pandemic anxiety, namely, fear and somatic concern. Quality sleep is often associated with reduced anxiety and enhanced psychological well-being. Thus, we also intend to study the role of sleep quality as a mediator between personality traits and the COVID-19 anxiety factors, i.e., fear and somatic concern, in the general adult population. The study aims to explore the extent to which the big five traits predict fear and somatic concern directly and indirectly through sleep quality. It was hypothesized that all five personality traits would predict fear and somatic concern directly and indirectly mediated through sleep quality. Therefore, a hypothetical model was proposed and tested. The model (Figure 1) included age, gender, and occupational status control variables.

## 2. Methodology

### 2.1. Study Design, Data Collection, and Procedures

A cross-sectional study design was followed for the research study. Data was collected using non-probability, convenience sampling methodology. Participation in the study was voluntary, and the participants were informed about the nature of the study. Furthermore, they were informed that they consented to participate in the study by filling the survey as per the Declaration of Helsinki (59th WMA General Assembly, 2009). No personally sensitive information was taken from the participants. The inclusion criteria in the study were that the participants should be of a minimum of 18 years of age, have completed a minimum of 12 years of schooling, and should be residing in India. A total of 308 participants completed the Google form consisting of the battery of questionnaires circulated through emails, WhatsApp, and Facebook during the period between 25 July 2021 to 20 September 2021. After the initial data screening, 12 incomplete forms were excluded. Excluding these 12 left a total sample of 296.

### 2.2. Measures for the Study

#### 2.2.1. Sociodemographic Information

Participants were asked to state their age, gender, occupation (employed or not), and highest educational qualification. The analysis included age, gender, and occupational status as control variables. The age of the participants ranged between 18 to 77 years, and the mean age was 32.57 years (SD = 14.92 years). Table 1 illustrates the sociodemographic profile of the sample.

#### 2.2.2. Big Five Inventory-10(BFI-10)

BFI-10 [55] is a short measure of personality traits. It has two items related to each of the five traits, i.e., Openness, Extraversion, Conscientiousness, Neuroticism, Agreeableness. The responses are on a 5-point rating scale, and a higher score indicates a high trait level. The authors have reported that the scale has good validity and reliability across the different samples. However, no Cronbach alpha was calculated as only two items per factor are present in the scale (e.g., Soto and John [56]).

#### 2.2.3. COVID-19 Pandemic Anxiety Scale (COVID-19 PAS)

COVID-19 PAS [57] is a 10-item short scale to assess the anxiety related to COVID-19 pandemic. A confirmatory factor analysis that tested the factor structure of COVID-19 PAS suggested a two-factor model with six items in factor one (fear) and four items in factor two (somatic concerns). The fear factor assessed the fear of going to markets, meeting strangers, listening to news updates, possible painful death due to the virus, and fear of lockdowns. The somatic concern factor assessed the perceived bodily concerns linked with COVID-19 (see Supplementary Figure S2). The higher scores on the scale indicated a high degree of COVID-19 anxiety. In addition, the scale’s Cronbach alpha (α = 0. 80) indicated good reliability for the scale. The Cronbach alpha for fear and somatic concern components was 0.78 and 0.82, respectively.

#### 2.2.4. Sleep Quality Scale (SQS)

SQS [58] was a single-item measure of sleep quality with strong reliability and validity. Sleep quality had to be rated from 0 to 10, with a higher rating indicating good sleep quality.

## 3. Results

### 3.1. Data Analysis

Statistical analyses have been conducted using SPSS version 21 and AMOS version 28. First, to determine the presence of common method bias, Herman’s single factor test was assessed. Next, t-test and bivariate correlations were computed using SPSS version 21. Cohen’s criteria were used to analyze and interpret the effect size of correlation coefficients [59]. Next, using AMOS 28, the multivariate normality of the data was assessed using Mardia’s coefficient [60,61]. The kurtosis coefficient of −2.982 with a critical ratio of 1.471 indicated multivariate normality. However, the skewness and kurtosis for individual study variables indicated univariate non-normality (Supplementary Materials, Table S2). Thus, path analysis using Maximum Likelihood (ML) estimation with bootstrapping (1000 resamples) was performed to generate accurate estimations of the regression coefficients with accompanying confidence intervals (bias-corrected at the 95% confidence level) and *p*-values [62,63]. Effects with *p* < 0.05 were considered statistically significant. Path analysis allows the assessment of the direct impact of independent variables on dependent variables and allows the studying of other relations like indirect and spurious. Further, it also enables comparison of the strength of relationships and identification of the mediators. Thus, path analysis was conducted to assess the effect of personality traits on COVID-19 pandemic anxiety factors, i.e., fear and somatic concern. Also, a mediational analysis was performed to examine the mediating role of sleep quality (mediator variable) between the big five personality traits (independent, exogenous variables) and fear (F) and somatic concerns (SC) (dependent, endogenous variables). In addition, age, gender, and occupational status were added to the model as control variables. To assess whether the drawn-out model from the bootstrapped sample provides model fit, χ^2^ and the Bollen–Stine bootstrap p [64] were assessed. Further, as per Hu and Bentler’s criteria, SRMR, RMSEA, and CFI were also assessed for model fit [65,66]. An excellent model fit is indicated by SRMR ≤ 0.08, RMSEA ≤ 0.06, and CFI ≥ 0.95.

### 3.2. Testing for Common Method Bias

Exploratory factor analysis of all the observed study variables using principal axis factoring indicated that the total variance explained by a single factor was 17.528%. This was significantly lower than the threshold of 50% (See Supplementary Table S1). Thus, it indicated no serious common method bias with the data.

### 3.3. Descriptive Analysis

Table 2 presents the means, standard deviations, and independent t-test results for the variables under study. Independent t-test results revealed significantly higher scores for women on neuroticism, fear, and somatic concerns. However, men had scored significantly higher on quality of sleep. However, because assessing gender differences was not an aim of this study and the number of participants was unsatisfactory to test the hypothesized model separately in men and women, all following analyses were conducted in the total sample [66]. Table 3 presents the bivariate correlation between all the study variables. All the correlations were below 0.50, indicating no strong relation between independent variables as per Cohen’s criteria [59].

### 3.4. Path Analysis: Direct and Indirect Associations

**Figure 2:**
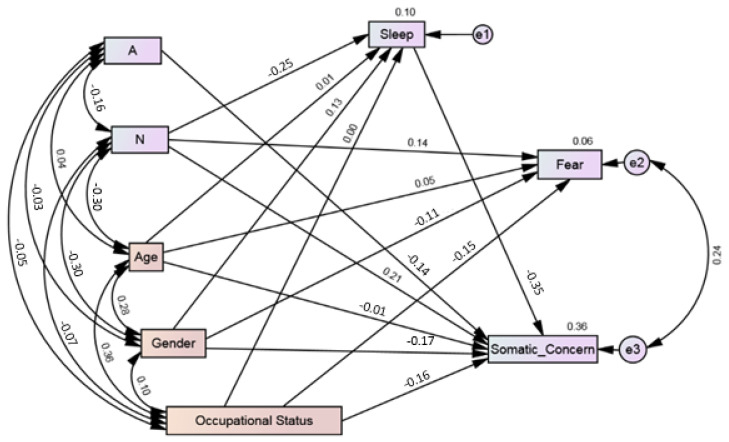
Path analysis model (M2) demonstrating the relationship between personality traits (A: Agreeableness; N: Neuroticism) and COVID-19 pandemic anxiety mediated through sleep quality. Note: The covariance between personality traits was assumed. Age, gender, and occupational status were included as control variables in the model.

The current investigation aimed to extend our understanding of vulnerability and protective factors to COVID-19 related anxiety. Thus, we tested a model. It was hypothesized that the personality traits of extraversion, openness to experience, conscientiousness, agreeableness, and neuroticism would directly affect the factors of COVID-19 anxiety, i.e., fear and somatic concern. Similarly, that sleep quality will mediate the relationship between personality traits and the factors of COVID-19 anxiety. The sociodemographic variables of gender, age, and occupational status were added to the models as control variables. Based on the hypothesis, a path model was developed. The resulting model (M1) was fully saturated (i.e., degree of freedom is zero) with 77 parameters. Fully saturated models always produce a perfect fit to the data. Therefore, model fit indices were neither examined nor reported. The model explained 7% of fear and 39% of somatic concern variance. In model 1, certain paths were not statistically significant, e.g., the direct relationship of extraversion on fear and somatic concern; the direct effect of openness on fear and somatic concern; the direct effect of agreeableness on fear; and the direct effect of neuroticism on fear (Table 4). Also, there was no significant effect of extraversion, openness, conscientiousness, and agreeableness on sleep. Similarly, sleep had no significant effect on fear (see Supplementary Figure S1). Thus, these non-significant paths were omitted one by one, and the model was recalculated. Age, gender, and occupational status remained in the model as control variables. The resulting (Figure 2) alternate model (M2) indicated that neuroticism had a significant direct effect on the factors of COVID-19 anxiety, i.e., fear (0.143, 95% CI (0.017, 0.247), *p* = 0.02). and somatic concern (0.213, 95% CI (0.099, 0.324), p = 0.003). Neuroticism had significant effect on quality of sleep (0.255, 95% CI (−0.364, −0.142), *p* = 0.003). Similarly, agreeableness had significant direct effect on somatic concern (−0.137, 95% CI (−0.235, −0.058), *p* = 0.002). Regarding mediation analysis, bootstrap indirect mediation analysis revealed a partial mediation by sleep quality in the relationship between neuroticism and somatic concerns (0.089, 95% CI (0.05, 0.14), *p* = 0.002). The resulting model indicated a non-significant chi-square test (χ2 = 4.547, *p* = 0.208, Bollenstine *p* = 0.226). The analysis of the indices indicates a good model fit (CMIN/DF = 1.516, RMSEA = 0.042 (95% CI, 0.000, 0.114), CFI = 0.995, TLI = 0.952, SRMR = 0.0209) as per the existing accepted criteria [64,65]. All the paths were statistically significant, and the model accounted for 6% of fear and 36% of somatic concerns. The mediator, sleep quality, also directly predicted decreased somatic concern (−0.351), indicating that somatic concerns decreased as sleep quality improved (Figure 2). However, there was no significant effect of sleep quality on fear (see Supplementary Tables S3–S10).

## 4. Discussion

The purpose of the current study was to explore the relationship between the big five personality traits and the factors of COVID-19 anxiety, i.e., fear and somatic concern. The study also explored whether sleep quality acts as a mediator determining the relationship between personality traits and COVID-19 anxiety. Our findings provide a preliminary indication regarding the relationship between these associations. The findings partially support the hypothesis that personality traits have a relationship with the factors of COVID-19 pandemic anxiety, and that quality of sleep has a mediator effect on mediating certain relationships.

As hypothesized in the conceptual model, neuroticism indicated significant effects on COVID-19 anxiety components of fear and somatic concerns. The personality trait of neuroticism is characterized by emotional instability, moodiness, irritability, and sadness. Thus, the results indicated that as these tendencies increased in individuals, COVID-19 anxiety components, i.e., fear and somatic concerns, also increased. COVID-19 anxiety in individuals is driven by the fear of getting infected by COVID-19, leading to a painful death. Thus, interacting with people and going to public places evokes fear. However, sleep quality only partially mediated the relationship between neuroticism and somatic concern. Expressly, the results indicated that the individuals who are higher on emotional instability report poor sleep quality and report more somatic concerns. Thus, it can also be mentioned that if the sleep quality enhances, somatic concern in the individuals can reduce. Somatic concern has been marked by excessive focus on physiological discomfort. Any slight physiological symptoms like palpitations, shortness of breath, fatigue, giddiness, or change in eating patterns make people hyper-vigilant. Thus, these individuals are forced to check and recheck to confirm the absence of perceived symptoms. Over-indulgence in safety behaviors has also created pressure in the daily life of individuals and disrupted daily functioning. Further fear of lockdowns has also enhanced fear and insecurity due to the lack of control over the situation.

In the current study, neuroticism is the only big five traits related to the COVID-19 pandemic anxiety factors, i.e., fear and somatic concern. Therefore, it aligns with the expected results as neuroticism is considered a risk factor for heightened anxiety. The current results are further corroborated by findings of the other previous research on general anxiety [8,17,21]. However, the other personality traits, except agreeableness, have not shown any significant relationship with pandemic anxiety, indicating that COVID-19 pandemic anxiety may be slightly different in its form and impact from the general anxiety.

The trait of agreeableness has shown a significant negative relationship with somatic concern. This indicates that individuals with agreeableness demonstrate less somatic concerns. In addition, agreeableness enables individuals to demonstrate higher trust, modesty, cooperation, and sympathy with others. These relationships possibly indicate that agreeableness functions as a protective personality trait that can buffer the impact of COVID-19 pandemic anxiety related to somatic concerns. Earlier research has also indicated that agreeableness makes people more adaptable to the new and changing environment [8].

The individuals scoring high on agreeableness tend to be more trusting and plausibly have faith that preventive measures will control the spread of the virus. Their faith and trust in others enhance their ability to deal with anxiety. Thus, they are better equipped to deal with the required changes in their lifestyle to match the sudden changes and the new normal created due to the COVID-19. The path diagram also indicates that sleep quality did not mediate the relationship with somatic concern for agreeableness trait. Similarly, agreeableness did not significantly affect fear directly or indirectly through the mediator of sleep quality.

Other personality traits, namely, extraversion, openness to experience, and conscientiousness, have not significantly influenced the factors of COVID-19 pandemic anxiety, namely fear and somatic concerns. This may be due to the different nature of the pandemic anxiety compared to general anxiety. The possible difference in both could be attributed to the definitive known source of pandemic anxiety compared to the unknown in the case of anxiety experienced by individuals diagnosed with anxiety disorders. People are anxious only when they fear getting infected by being in unknown places outside their secure bio-bubble. Anxiety is usually pervasive and cannot be zeroed down to any specific source. In the case of pandemic anxiety, there is a known source of the infection from the coronavirus (SARS-COV-2). Thus, due to this discrete nature of anxiety, the other personality traits may not have influenced the experience of pandemic anxiety. People who are high on neuroticism are more inclined to be negatively influenced by anxiety arising from the two factors. Other individuals can handle the pandemic anxiety as they are only required to follow the protective measures, and the fear and related somatic concern can be managed.

The present study results further revealed that of the big five personality traits, the factor of fear had been predicted only by neuroticism out of the four traits. A pandemic like COVID-19, which affects various parts of the world with differing intensities across time, spreads in waves regarding the number of people infected with the virus. Thus, the fear component may vary across time. As the number of COVID-19 cases rises, the fear also rises. There are often restrictions, like lockdown which enhance fear among people. However, lockdown cannot be imposed indefinitely. The adverse economic impact on nations and individuals can be immense. Thus, we have seen that as the number of COVID-19 infections goes down, the restrictions are lifted. With the easing of restriction, the fear component also reduces. However, as the virus has frequently mutated, the world needs to be vigilant.

The current data was collected during July-September 2021 when the cases were waning in India after reaching peaks during the devastating second wave of infections during May 2021. Therefore, the fear may have probably waned due to the decrease in the positivity rate of COVID-19. Thus, the study’s findings can be explained considering the changed scenario. Another plausible reason could be that as the pandemic has become so pervasive, people have started to accept it as a part of their life, and thus, it may be ignored consciously. Furthermore, the coverage of news related to the status of COVID-19 in media has also reduced a bit. Thus, it may not arouse anxiety in people compared to earlier times when there was a continuous bombardment of coronavirus-related news. Thus, it may be presumed that these factors may have reduced fear in people. However, longitudinal studies are needed to assess these plausible explanations.

## 5. Limitations

The study has some limitations, and the conclusions should be considered in light of these limitations. Firstly, there are methodological limitations that are prevalent in the field of psychology as a whole. Non-probability sampling and a cross-sectional design were used in this study which restricts causal inferences. In addition, the variables like age, gender, and occupational status (covariates) were controlled. However, theoretical evidence suggests that results may be influenced. Similarly, only the role of sleep quality as a mediator was investigated in the study. However, the role of other potential mediators like coping, resilience, and spirituality have not been assessed. Secondly, the participants in this study may not have been truly representative of the general population as more than 50% were females, and a more equally numbered male sample may have been better. Further, separate models for males and females were not assessed. Thirdly, the data collected using self-report measures may be subjected to social desirability and self-report errors.

Additionally, the data was collected when cases were on the decline. Thus, there is a need for testing the conclusions again as the sample was also not very large. Lastly, the BFI-10 personality measure was used in the study whose perceived reliability is considered low compared to the available similar longer measures. Thus, future studies may be needed to validate further and confirm these findings.

## 6. Conclusions

Despite the above limitations, the current study expands our understanding of vulnerability and protective factors regarding COVID-19 anxiety. Contrary to our perception, the path analysis results showed that sleep quality emerged as a partial mediator for neuroticism’s influence on the somatic concern. Similarly, only the trait of neuroticism, whether directly and indirectly, affected the COVID-19 pandemic anxiety factors, i.e., fear and somatic concern. Additionally, only agreeableness has emerged as a protective factor in the case of COVID-19 pandemic anxiety. The present study findings may have important implications for dealing with pandemic anxiety. The derived model indicates that good sleep quality can reduce somatic concern in people experiencing anxiety, particularly those who are higher on neuroticism. Notably, it seems that people high on neuroticism are predisposed to experience more fear and somatic concerns. However, people high on agreeableness seem to have fewer somatic concerns. Thus, the current study’s findings also provide a preliminary indication that the relationship of big five personality traits with pandemic anxiety might be quite different from the existing relationship with general anxiety. Thus, the findings of the COVID-19 anxiety studies using general anxiety instruments have to be cautiously interpreted when used for assessing pandemic anxiety.

Further, the current COVID-19 pandemic has ravaged the planet for two years. As the virus SARS-COV-2 is constantly mutating and with the emergence of mutations like Omicron predicted to be highly transmissible, pandemic anxiety may remain high even in the coming times. With no sign of an early end to the pandemic, mental health professionals should develop intervention programs to enhance sleep quality so that the somatic concerns arising from pandemic anxiety can be better managed. Thus, the intensity of anxiety perceived by individuals may be managed with sleep-based intervention programs. Intervention strategies for improvement in sleep quality can prepare individuals for combating pandemic anxiety and fatigue developed and driven environment by the persistent long-term nature of the pandemic. However, the current findings are based on a single male and female model. As gender can play a significant role in determining these relationships, future studies using larger samples are recommended to develop separate models for males and females to explore the relationships further.

Nevertheless, the current study presents preliminary evidence demonstrating the model explaining the relationship between personality traits, sleep quality, and COVID-19 pandemic anxiety. The current study helps mental health professionals better understand the role of personality traits in determining COVID-19 anxiety and demonstrates how sleep quality mediates the relationship, and enables them to develop psychological interventions to reduce anxiety and distress among individuals for the current and future pandemics.

## Figures and Tables

**Table 1:** Sociodemographic profile of the sample.

S. No.	Socio-Demographic Variable		*N*	Percentage
1.	Gender	Females	183	61.80
		Males	113	38.20
2.	Marital Status	Married	149	50.34
		Unmarried	138	46.62
		Others	9	3.04
3.	Education	Higher secondary	74	25.00
		Undergraduate	7	2.360
		Graduate	103	34.80
		Postgraduate	100	33.78
		PhD	12	4.05
4.	Occupational Status	Not employed	168	56.76
		Employed	129	43.24

**Table 2:** Means (M), standard deviations (SD), and independent sample t-tests on all measures.

	Total		Females		Males		t	*p*
Variable	(*N* = 296)		(*n* = 183)		(*n* = 113)			
	M	SD	M	SD	M	SD		
Extraversion	6.35	2.23	6.17	2.41	6.65	1.88	−1.77	0.078
Conscientiousness	7.16	1.73	7.02	1.65	7.39	1.82	−1.79	0.075
Neuroticism	5.95	2.13	6.44	2.17	5.15	1.81	5.30	0.000
Openness	6.79	1.56	6.79	1.63	6.79	1.43	0.00	0.997
Agreeableness	7.76	1.71	7.80	1.71	7.70	1.70	0.51	0.611
Sleep	6.80	2.34	6.43	2.45	7.42	2.01	−3.60	0.000
Fear	5.90	3.62	6.33	3.58	5.20	3.59	2.62	0.009
Somatic Concern	2.35	2.54	2.99	2.79	1.32	1.61	5.80	0.000

**Table 3:** Correlations for the study variables.

Variables	1	2	3	4	5	6	7	8
Extraversion (1)	1							
Conscientiousness (2)	0.243 **	1						
Neuroticism (3)	−0.252 **	−0.327 **	1					
Openness (4)	−0.002	−0.067	0.009	1				
Agreeableness (5)	0.186 **	0.053	−0.163 **	0.045	1			
Sleep (6)	0.146 *	0.146 *	−0.295 **	0.052	0.083	1		
Fear (7)	−0.046	−0.074	0.171 **	−0.021	−0.116 *	−0.091	1	
Somatic Concern (8)	−0.244 **	−0.268 **	0.401 **	−0.076	−0.208 **	−0.470 **	0.318 **	1

Note: ** *p* < 0.01; * *p* < 0.01.

**Table 4:** Standardized effects and corresponding 95% bias-corrected bootstrap CIS for model 1.

Model Pathways	Direct				Indirect via Sleep				Total Effects			
	(95% CI)				(95% CI)				(95% CI)			
	β	Lower	Upper	*p*	β	Lower	Upper	*p*	β	Lower	Upper	*p*
E → F	0.013	−0.096	0.136	0.747	−0.001	−0.022	0.007	0.496	0.012	−0.102	0.130	0.791
O → F	−0.022	−0.157	0.088	0.627	−0.001	−0.018	0.006	0.451	−0.023	−0.163	0.088	0.608
C→ F	−0.014	−0.130	0.117	0.845	−0.001	−0.016	0.007	0.561	−0.016	−0.130	0.109	0.813
A→ F	−0.109	−0.213	0.011	0.071	−0.001	−0.017	0.005	0.546	−0.110	−0.215	0.010	0.065
N → F	0.115	−0.010	0.232	0.081	0.005	−0.028	0.036	0.723	0.121	−0.003	0.236	0.055
E → SC	−0.086	−0.181	0.021	0.095	−0.020	−0.072	0.019	0.299	−0.106	−0.209	0.009	0.068
O → SC	−0.068	−0.172	0.023	0.130	−0.019	−0.060	0.020	0.340	0.086	−0.194	0.011	0.083
C→ SC	−0.109	−0.214	0.007	0.062	−0.017	−0.064	0.027	0.390	−0.126	−0.234	−0.011	0.029
A → SC	−0.142	−0.245	−0.050	0.004	−0.012	−0.049	0.024	0.541	−0.154	−0.262	−0.051	003
N → SC	0.167	0.057	0.289	0.007	0.075	−0.038	0.124	0.002	0.243	0.131	0.361	0.001

Note: E: Extraversion, O: Openness, C: Conscientiousness, A: Agreeableness, N: Neuroticism; F: Fear, SC: Somatic Concern.

## Data Availability

Data available upon request from the corresponding author.

## Supplementary Materials

The following are available online at https://www.mdpi.com/article/10.3390/bs12020024/s1; Figure S1: Results of mediation path analysis (Model M1) showing the relationship among the Big Five personality domains and COVID-19 anxiety components; Figure S2: Confirmatory Factor analysis for the two factor COVID-19 pandemic anxiety scale; Table S1: Herman single factor test results, Table S2: Assessment of normality; Table S3: Standardized total effects for Model 1; Table S4: Standardized direct effects for Model 1; Table S5: Standardized indirect effects for Model 1; Table S6: Model Fit Summary for Model 1; Table S7: Standardized total effects for Model 2; Table S8: Standardized direct effects for Model 2; Table S9: Standardized indirect effects for Model 2; Table S10: Model fit summary for Model 2.
