# Supplementary material for: The Relationship between Personality Traits and COVID-19 Anxiety: A Mediating Model
Source: Behav Sci (Basel). 2022 Jan 26;12(2):24. doi: 10.3390/bs12020024 (PMC8869446; doi:10.3390/bs12020024)
Supplement: Supplementary file 1 [file behavsci-12-00024-s001.zip › Supplementary Figures.pdf]

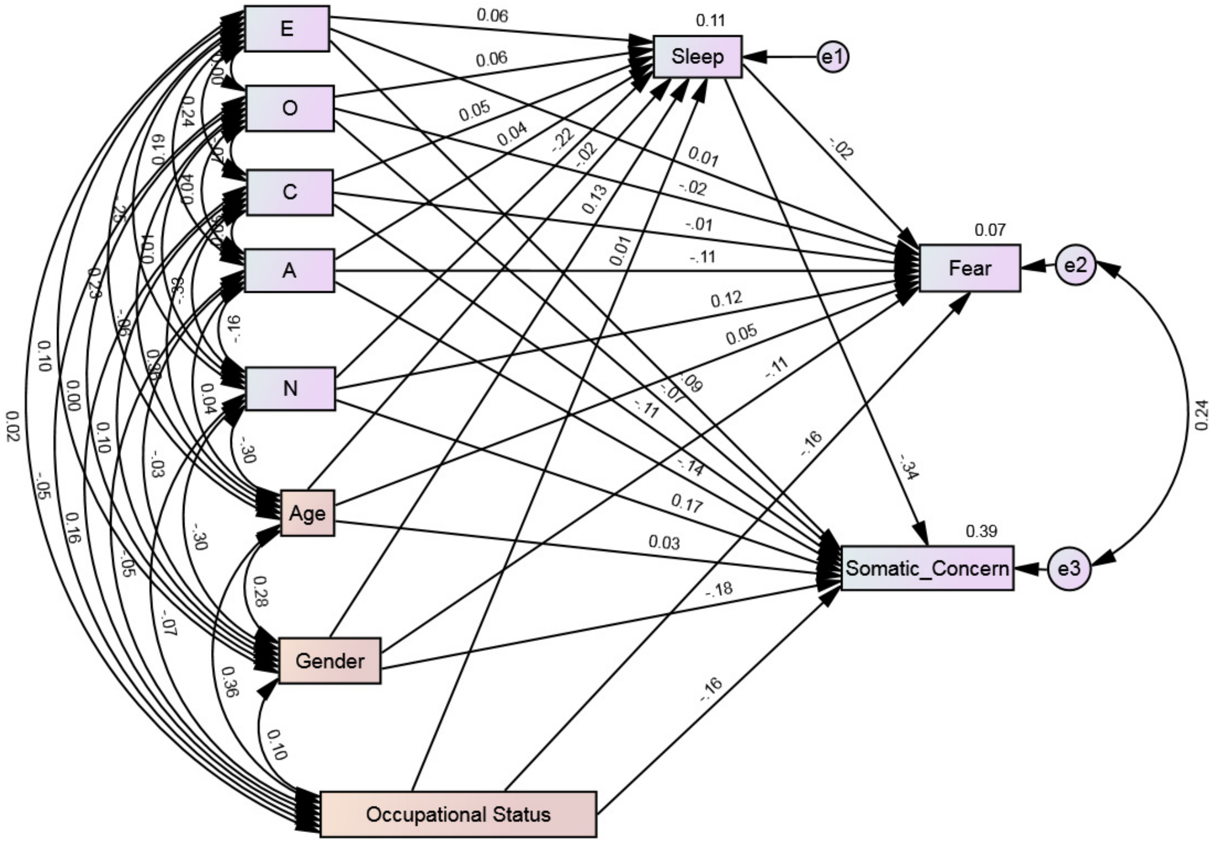

Figure S1. Results of mediation path analysis (Model M1) showing the relationship among the Big Five personality domains (E = Extraversion, O: Openness, C: Conscientiousness, A: Agreeableness, N: Neuroticism) and COVID 19 anxiety components (Fear and Somatic Concern).

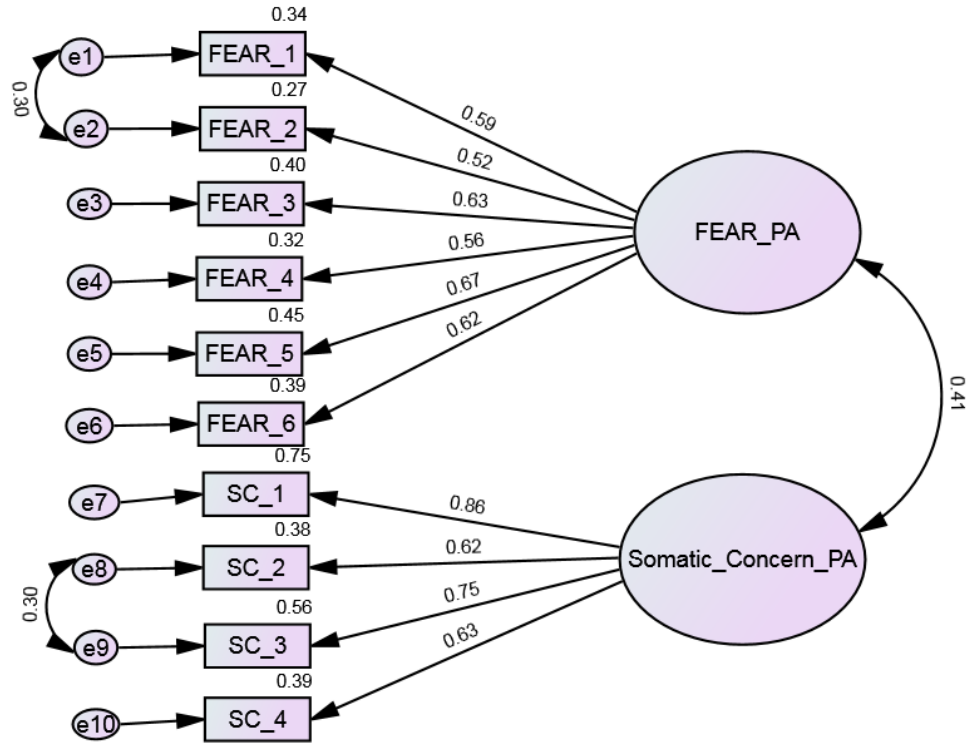

Figure S2. Confirmatory Factor analysis (N=296) for the two factor COVID 19 pandemic anxiety scale ($\chi 2$ = 70.415, CMIN/DF=2.200, RMSEA: 0.064, GFI: 0.958, CFI: 0.957, TLI: 0.939, SRMR:.0534).
