# Supplementary material for: The Relationship between Personality Traits and COVID-19 Anxiety: A Mediating Model
Source: Behav Sci (Basel). 2022 Jan 26;12(2):24. doi: 10.3390/bs12020024 (PMC8869446; doi:10.3390/bs12020024)
Supplement: Supplementary file 1 [file behavsci-12-00024-s001.zip › Supplementary tables.pdf]

Table S1. Herman single factor test results

Factor	Initial Eigenvalues			Extraction Sums of Squared Loadings		
	Total	% of Variance	Cumulative %	Total	% of Variance	Cumulative %
1	4.387	20.889	20.889	3.681	17.528	17.528
2	2.388	11.370	32.259			
3	1.515	7.215	39.475			
4	1.294	6.164	45.639			
5	1.238	5.895	51.534			
6	1.108	5.276	56.810			
7	1.020	4.857	61.667			
8	0.897	4.273	65.939			
9	0.833	3.969	69.908			
10	0.769	3.661	73.569			
11	0.758	3.611	77.180			
12	0.684	3.257	80.437			
13	0.620	2.953	83.390			
14	0.603	2.871	86.261			
15	0.557	2.654	88.915			
16	0.486	2.312	91.227			
17	0.443	2.111	93.338			
18	0.433	2.062	95.400			
19	0.377	1.793	97.194			
20	0.322	1.534	98.728			
21	0.267	1.272	100.000			

Note: Extraction Method: Principal Axis Factoring

Table S2. Assessment of normality (N=296)

Variable	min	max	skew	c.r	kurtosis	c.r
Age	18.000	77.000	0.864	6.069	-0.333	-1.169
Gender	0.000	1.000	0.487	1.916	-1.763	-6.762
Occupation	0.000	1.000	0.273	3.419	-1.926	-6.192
E	2.000	10.000	-0.133	-0.931	-0.87	-3.057
O	3.000	10.000	0.200	1.404	-0.105	-0.367
C	3.000	10.000	0.010	0.072	-0.635	-2.232
A	2.000	10.000	-0.538	-3.781	-0.15	-0.525
N	2.000	10.000	-0.062	-0.438	-0.688	-2.415
Sleep	0.000	10.000	-0.770	-5.409	0.08	0.283
Fear	0.000	18.000	0.477	3.35	-0.073	-0.258
Somatic_Concern	0.000	11.000	1.239	8.702	0.881	3.094
Multivariate					-2.892	-1.471

Table S3. Standardized total effects for Model 1

lower bounds (BC)									
	Age	Gender	Occupation	O	E	C	N	A	Sleep
Sleep	-0.135	0.035	-0.112	-0.058	-0.058	-0.084	-0.337	-0.074	0.000
Fear	-0.068	-0.228	-0.277	-0.163	-0.102	-0.130	-0.003	-0.215	-0.141
Somatic_Concern	-0.076	-0.307	-0.250	-0.194	-0.209	-0.234	0.131	-0.262	-0.443
**upper bounds (BC)**									
	Age	Gender	Occupation	O	E	C	N	A	Sleep
Sleep	0.114	0.248	0.115	0.167	0.201	0.174	-0.105	0.142	0.000
Fear	0.170	0.004	-0.051	0.088	0.130	0.109	0.236	0.010	0.121
Somatic_Concern	0.134	-0.136	-0.060	0.011	0.009	-0.011	0.361	-0.051	-0.232
**Standardized total effects - two tailed significance (BC)**									
	Age	Gender	Occupation	O	E	C	N	A	Sleep
Sleep	0.854	0.005	0.908	0.368	0.304	0.407	0.002	0.548	...
Fear	0.471	0.061	0.011	0.608	0.791	0.813	0.055	0.065	0.765
Somatic_Concern	0.480	0.002	0.006	0.083	0.068	0.029	0.001	0.003	0.003

Table S4. Standardized direct effects for Model 1

lower bounds (BC)									
	Age	Gender	Occupation	O	E	C	N	A	Sleep
Sleep	-0.135	0.035	-0.112	-0.058	-0.058	-0.084	-0.337	-0.074	0.000
Fear	-0.070	-0.225	-0.275	-0.157	-0.096	-0.130	-0.010	-0.213	-0.141
Somatic_Concern	-0.066	-0.265	-0.242	-0.172	-0.181	-0.214	0.057	-0.245	-0.443
**upper bounds (BC)**									
	Age	Gender	Occupation	O	E	C	N	A	Sleep
Sleep	0.114	0.248	0.115	0.167	0.201	0.174	-0.105	0.142	0.000
Fear	0.170	0.007	-0.049	0.088	0.136	0.117	0.232	0.011	0.121
Somatic_Concern	0.123	-0.098	-0.060	0.023	0.021	0.007	0.289	-0.050	-0.232
**Standardized direct effects for Model 1 - two tailed significance (BC)**									
	Age	Gender	Occupation	O	E	C	N	A	Sleep
Sleep	0.854	0.005	0.908	0.368	0.304	0.407	0.002	0.548	...
Fear	0.482	0.055	0.012	0.627	0.747	0.845	0.081	0.071	0.765
Somatic_Concern	0.476	0.002	0.003	0.130	0.095	0.062	0.007	0.004	0.003

Table S5. Standardized indirect effects for Model 1

lower bounds (BC)									
	Age	Gender	Occupation	O	E	C	N	A	Sleep
Sleep	0.000	0.000	0.000	0.000	0.000	0.000	0.000	0.000	0.000
Fear	-0.008	-0.026	-0.012	-0.018	-0.022	-0.016	-0.028	-0.017	0.000
Somatic_Concern	-0.040	-0.097	-0.040	-0.060	-0.072	-0.064	0.038	-0.049	0.000
**upper bounds (BC)**									
	Age	Gender	Occupation	O	E	C	N	A	Sleep
Sleep	0.000	0.000	0.000	0.000	0.000	0.000	0.000	0.000	0.000
Fear	0.015	0.015	0.006	0.006	0.007	0.007	0.036	0.005	0.000
Somatic_Concern	0.049	-0.011	0.040	0.020	0.019	0.027	0.124	0.024	0.000
**Standardized indirect effects for Model 1 - two tailed significance (BC)**									
	Age	Gender	Occupation	O	E	C	N	A	Sleep
Sleep	...	...	...	...	...	...	...	...	...
Fear	0.720	0.623	0.680	0.451	0.496	0.561	0.723	0.546	...
Somatic_Concern	0.846	0.005	0.916	0.340	0.299	0.390	0.002	0.541	...

Table S6. Model Fit Summary for Model 1

CMIN					
Model	NPAR	CMIN	DF	P	CMIN/DF
Default model	77	0	0		
Saturated model	77	0	0		
Independence model	22	448.572	55	0	8.156
**Baseline Comparisons**					
Model	NFI Delta1	RFI rho1	IFI Delta2	TLI rho2	CFI
Default model	1		1		1
Saturated model	1		1		1
Independence model	0	0	0	0	0
**RMSEA**					
Model	RMSEA	LO 90	HI 90	PCLOSE	
Independence model	0.156	0.143	0.169	0	

Table S7. Standardized total effects for Model 2

lower bounds (BC)						
	Age	Gender	Occupation	N	Sleep	A
Sleep	-0.101	0.026	-0.128	-0.364	0.000	0.000
Fear	-0.076	-0.219	-0.270	0.017	0.000	0.000
Somatic_Concern	-0.128	-0.309	-0.246	0.197	-0.455	-0.235
**upper bounds (BC)**						
	Age	Gender	Occupation	N	Sleep	A
Sleep	0.125	0.243	0.106	-0.142	0.000	0.000
Fear	0.161	0.015	-0.042	0.247	0.000	0.000
Somatic_Concern	0.094	-0.128	-0.050	0.413	-0.249	-0.058

Standardized total effects for Model 2 - two tailed significance (BC)

	Age	Gender	Occupation	N	Sleep	A
Sleep	0.892	0.009	0.963	0.002	...	...
Fear	0.513	0.069	0.012	0.020	...	...
Somatic_Concern	0.798	0.002	0.007	0.002	0.003	0.002

Table S8. Standardized direct effects for Model 2

lower bounds (BC)						
	Age	Gender	Occupation	N	Sleep	A
Sleep	-0.101	0.026	-0.128	-0.364	0.000	0.000
Fear	-0.076	-0.219	-0.270	0.017	0.000	0.000
Somatic_Concern	-0.115	-0.257	-0.244	0.099	-0.455	-0.235
**upper bounds (BC)**						
	Age	Gender	Occupation	N	Sleep	A
Sleep	0.125	0.243	0.106	-0.142	0.000	0.000
Fear	0.161	0.015	-0.042	0.247	0.000	0.000
Somatic_Concern	0.085	-0.085	-0.050	0.324	-0.249	-0.058

Standardized direct effects for Model 2 - two tailed significance (BC)

	Age	Gender	Occupation	N	Sleep	A
Sleep	0.892	0.009	0.963	0.002	...	...
Fear	0.513	0.069	0.012	0.020	...	...
Somatic_Concern	0.805	0.002	0.005	0.003	0.003	0.002

Table S9. Standardized indirect effects for Model 2

lower bounds (BC)						
	Age	Gender	Occupation	N	Sleep	A
Sleep	0.000	0.000	0.000	0.000	0.000	0.000
Fear	0.000	0.000	0.000	0.000	0.000	0.000
Somatic_Concern	-0.046	-0.099	-0.037	0.050	0.000	0.000
Standardized indirect effects for Model 2 - upper bounds (BC)						
	Age	Gender	Occupation	N	Sleep	A
Sleep	0.000	0.000	0.000	0.000	0.000	0.000
Fear	0.000	0.000	0.000	0.000	0.000	0.000
Somatic_Concern	0.038	-0.009	0.044	0.140	0.000	0.000
Standardized indirect effects for Model 2 - two tailed Significance (BC)						
	Age	Gender	Occupation	N	Sleep	A
Sleep	...	...	...	...	...	...
Fear	...	...	...	...	...	...
Somatic_Concern	0.892	0.008	0.967	0.002	...	...

Table S10. Model fit summary for Model 2

Model	NPAR	CMIN	DF	P	CMIN/DF
Default model	41	4.547	3	0.208	1.516
Saturated model	44	0.000	0		
Independence model	16	329.380	28	0.000	11.764
Model	NFI Delta1	RFI rho1	IFI Delta2	TLI rho2	CFI
Default model	0.986	0.871	0.995	0.952	0.995
Saturated model	1.000		1.000		1.000
Independence model	0.000	0.000	0.000	0.000	0.000
Model	RMSEA	LO 90	HI 90	PCLOSE	
Default model	0.042	0.000	0.114	0.477	
Independence model	0.191	0.173	0.210	0.000	
